# Obesity, physical activity, and gene–environment interactions: a natural experiment framework

**DOI:** 10.1186/s41043-026-01312-y

**Published:** 2026-04-09

**Authors:** Lei Liu, Xuxiu Zhuang, Haonan Zhou, Yanan Ma, Gengrun Sun, Yang Liu, Deliang Wen

**Affiliations:** 1 Health Sciences Institute, China Medical University, Shenyang, 110122 Liaoning Province PR China; 2 School of Life Sciences and Health, University of Health and Rehabilitation Sciences, Qingdao, 266113 Shandong Province PR China; 3 Key Laboratory of Obesity and Glucose/Lipid Associated Metabolic Diseases, China Medical University, Shenyang, 110122 Liaoning Province PR China; 4 Endocrinology Department, Qingdao Hospital, University of Health and Rehabilitation Sciences (Qingdao Municipal Hospital), Qingdao, 266113 Shandong Province PR China

**Keywords:** Overweight and obesity, Children and adolescents, Physical activity, Genetic susceptibility, Gene–environment interaction

## Abstract

**Background:**

Obesity results from the interaction of polygenic susceptibility and environmental factors. Given this complex etiology, physical activity (PA) remains a cornerstone of cost-effective intervention strategies. This longitudinal natural experiment investigated how PA modifies the effects of genetic predisposition on obesity in Chinese youth.

**Methods:**

We conducted a 4-year natural experiment leveraging curriculum-driven PA disparities in a specialized arts school (*n* = 591), creating distinct high-PA (HPA) and low-PA (LPA) exposure groups. Weighted genetic risk scores (WGRSs) were calculated from 13 Asian-derived obesity-related single-nucleotide polymorphisms. Annual anthropometric, metabolic, and lifestyle data were analyzed using generalized linear mixed models to assess gene–PA interactions on obesity.

**Results:**

The WGRS predicted baseline obesity measures, with each unit increase associated with a 0.21-kg/m² higher BMI. Over the natural experiment period, BMI increases in the HPA group were smaller than in the LPA group. After adjusting for age, sex, ethnicity, and dietary factors, significant WGRS–PA interactions were observed for BMI trajectories. Participants with higher genetic risk for obesity experienced greater BMI and weight reduction benefits from sustained long-term PA.

**Conclusions:**

In summary, the present study identified a significant interaction effect between PA levels and WGRS in modifying BMI trajectories. Genetic susceptibility significantly modifies the protective effects of long-term PA on BMI progression in this cohort of Chinese youth.

**Supplementary Information:**

The online version contains supplementary material available at 10.1186/s41043-026-01312-y.

## Background

The global prevalence of overweight and obesity has risen sharply, making it a critical public health challenge due to its substantial contribution to morbidity and premature mortality [1]. This pandemic is closely tied to modern lifestyle changes, particularly declining physical activity (PA) levels, which disrupt energy homeostasis and promote a positive energy balance [2]. Increased PA is a safe and effective means of energy expenditure, serving both as a therapeutic intervention for obesity-related comorbidities and as a modulator of aging trajectories, functional capacity, and healthspan [3]. Longitudinal epidemiological evidence shows that sustained PA during developmental years confers lifelong cardiometabolic benefits [4]. Systematic reviews by Jakicic et al. [5]. have demonstrated that PA interventions alone can produce significant weight loss—on average 2–3 kg over periods of 6 months or more—accompanied by reductions in total and visceral adiposity.

Previous research on the relationship between obesity-associated genetic variants and longitudinal weight changes has produced heterogeneous and sometimes inconsistent findings [6–8]. A meta-analysis of 218,166 adults and 19,268 children/adolescents found a significant interaction between the *FTO* rs9939609 polymorphism and PA on obesity risk in adults, although this interaction was absent in children/adolescents [9]. Similarly, Wang et al. [10]. analyzing 20-year body mass index (BMI) trajectories in the Nurses’ Health Study (NHS) and Health Professionals Follow-Up Study (HPFS) cohorts, observed a significant interaction between genetic susceptibility and PA levels on BMI trajectories. For every 10 additional risk alleles, the highest PA group experienced a BMI change of − 0.02 kg/m^2^ over 4 years, compared with + 0.24 kg/ m^2^ in the lowest PA group. These findings suggest that individuals with a high genetic predisposition to obesity may gain greater benefits from increased PA. If genetic susceptibility data can be used to identify and differentiate the degree of responsiveness to PA interventions, such information could be of great public health value in developing precision intervention program.

Natural experiments are observational studies in which exposure allocation occurs through mechanisms, outside the investigator’s control, allowing for causal inference when experimental randomization is not feasible. This approach has historically enabled landmark epidemiological discoveries, such as John Snow’s identification of cholera transmission pathways [11] and investigations into the long-term metabolic consequences of the 1959–1961 Chinese famine [12]. In obesity research, natural experiments have become an increasingly common framework for assessing the effects of policies, programs, and built environment modifications on obesity prevention and control. Bennett et al. [13]. systematically reviewed 156 natural experiments evaluating obesity-related outcomes across domains including PA, dietary behaviors, sugar-sweetened beverage consumption, and healthcare policies, providing robust evidence to inform prevention strategies. Differences in PA levels among arts students, driven by curriculum requirements [14], offer a strong natural experiment framework for investigating gene–environment interactions in obesity through distinct patterns of exposure.

Current evidence on gene–PA interactions in obesity pathogenesis comes largely from cross-sectional studies and short-term interventions, which have limited generalizability across populations and over time. To address these limitations, we applied a natural experiment framework by longitudinally tracking an arts student cohort. Therefore, this longitudinal natural experiment was designed to address the following research question: Among Chinese youth, does the long-term effect of PA on obesity-related outcomes exhibit a gene-PA interaction? We hypothesize that such an interaction exists, and that the protective effect of PA against BMI gain would be most pronounced among individuals with a high genetic susceptibility to obesity. This approach aims to advance personalized obesity prevention strategies informed by genetic susceptibility.

## Methods

### Ethics statement

This study was approved by the ethics committee of the China Medical University (Ethics Approval No. [2020] 077). Each participant and their parents or legal guardians signed an informed consent form. All methods were performed in accordance with the Declaration of Helsinki and relevant guidelines.

### Study design and participants

**Fig. 1:**
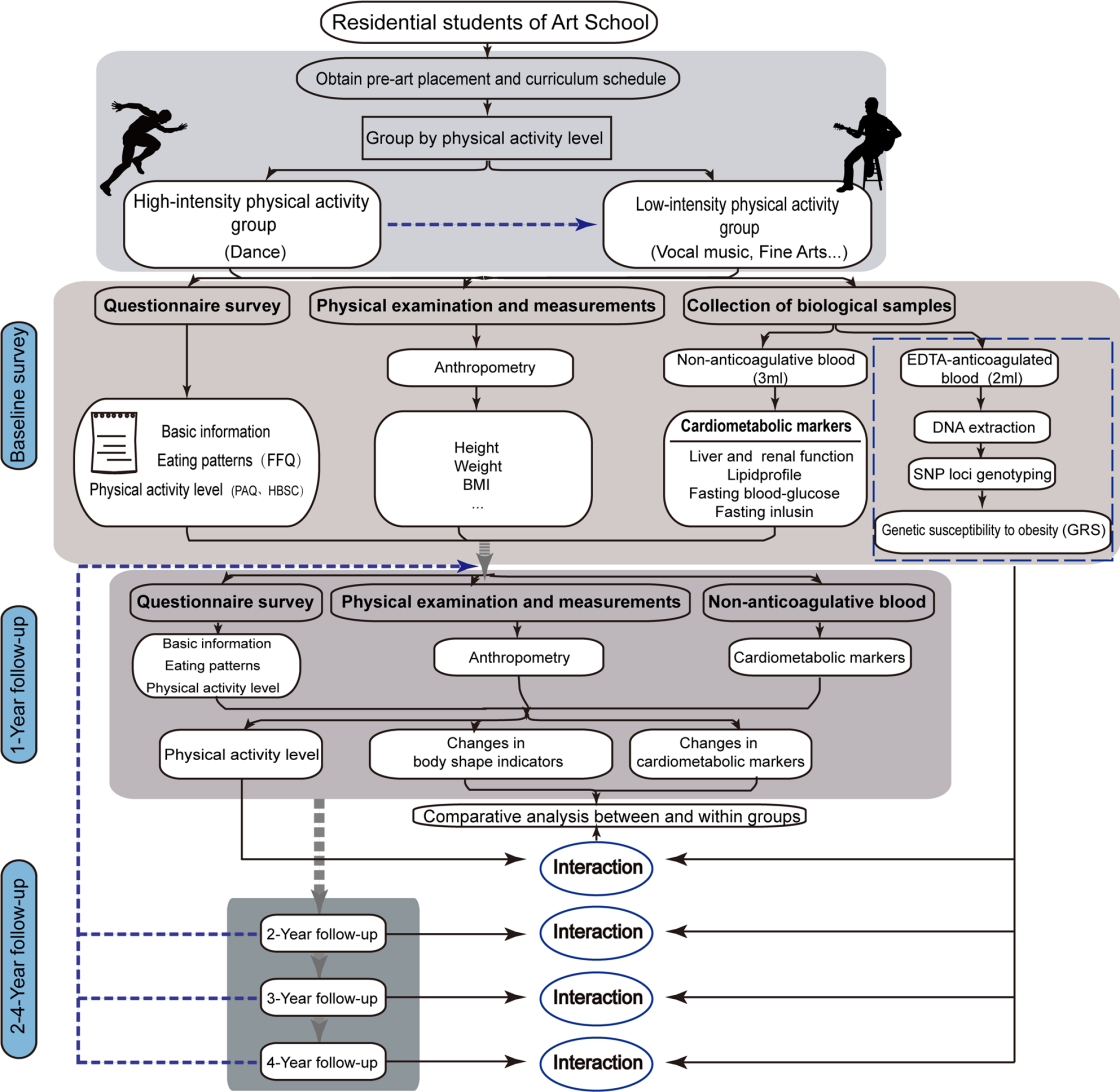
Schematic diagram of the research design flowchart

This study, targeted full-time art students enrolled in a 9-year consistent art school in Shenyang City. The study was conducted from September 2015 to September 2019, encompassing a total follow-up period of 4 years. Enrollment and follow-up investigations were conducted at the start of each new academic year. For the current analysis, 519 students who met the following eligibility criteria were included: initiation of full-time specialized arts training; absence of secondary obesity caused by metabolic disorders, congenital genetic diseases, or endocrine conditions; willingness to undergo anthropometric assessments and complete study questionnaires; and no anticipated relocation or school transfer during the follow-up period. As illustrated in Fig. 1 and Figure S1, 90 students enrolled in 2015 were followed for 4 years; 68 students enrolled in 2016 were followed for 3 years; 162 students enrolled in 2017 were followed for 2 years; and 271 students enrolled in 2018 were followed for 1 year. Consequently, a total of 591 participants completed at least 1 year of follow-up, 320 completed at least 2 years, 158 completed at least 3 years, and 90 completed the full 4 years of the natural experiment.

### Grouping of study participants

Based on their enrolled discipline at baseline, the participants were primarily grouped into four specialized disciplines: dance, music, fine arts, and film/television. According to Ainsworth et al. [15]. in the *Compendium of Physical Activities*, dance disciplines have mean intensities of 4.5–7.3 metabolic equivalents (METs), classifying them as high-intensity PA (HPA). By contrast, music, fine arts, and film/television disciplines display lower PA levels (1.8–2.0 METs), corresponding to low-intensity PA (LPA). Using these criteria, the participants were stratified into two groups.

### Data collection and diagnostic criteria of overweight and obesity

Height and body weight were measured using a wall-mounted medical stadiometer and a calibrated electronic scale (Seca GmbH, Hamburg, Germany), with participants barefoot and wearing light clothing, following standardized anthropometric protocols. BMI was calculated as weight (kg) divided by height (m) squared. Overweight and obesity were defined according to the criteria for Chinese children and adolescents established by the Working Group on Obesity in China, with BMI cut-offs specific to age and sex (Table S1, see Additional file 1).

### Questionnaire survey

Basic demographic information—including class, sex, age, birthdate, ethnicity, and art specialization—was collected using structured questionnaires. PA levels were assessed with the validated Health Behaviors in School-aged Children (HBSC) questionnaire, which includes four items evaluating both habitual and recent (past-week) PA patterns. Dietary intake was assessed using a non-quantitative food frequency questionnaire (FFQ) covering consumption frequencies across 24 food categories (Table S2, see Additional file 1). Dietary patterns were then derived through exploratory factor analysis to identify latent food intake profiles. All questionnaires were administered under the supervision of trained investigators to ensure adherence to protocol and data reliability.

### Blood samples collection, treatment and biochemical measurements

Antecubital venous blood samples were collected from all participants by professional nurses in the school medical room. The procedures of blood samples collection, treatment and biochemical measurements were performed in accordance with our previously established methods [16].

### Selection of trait-increasing alleles, genotyping, genotyping, and weighted genetic risk score (WGRS) calculation

Genomic DNA extraction, selection of obesity-associated single-nucleotide polymorphisms (SNPs), and genotyping of targeted SNPs followed the methodologies described in our prior work [16]. The SNPs were successfully genotyped in all participants, with interpolated controls showing 99% concordance across all loci. All SNPs conformed to Hardy–Weinberg equilibrium. One BMI susceptibility gene, *TFAP2B* rs471521, was excluded because of a lower genotyping success rate. The detection response rates for the remaining 13 SNPs were all above 98%. Effect allele frequencies and call rates for each SNP are presented in Table S3 (see Additional file 1). Using these 13 SNPs, we assumed each locus contributed independently in an additive manner. To account for the effect size of each trait-increasing allele, we calculated the WGRS as: WGRS = (β_1_ × SNP_1_ + β_2_ × SNP_2_ + … + β_n_ × SNP_n_) × (total number of SNPs /sum of the β coefficients), where β represents the coefficient of each SNP for higher BMI level as reported in genome-wide association study (GWAS), and SNP_1_, SNP_2_ … SNP_n_ indicate the number of trait-increasing alleles (0, 1, or 2) for each locus. A higher WGRS indicates greater genetic risk for higher BMI.

### Statistical analysis

Continuous variables were reported as the mean ± standard deviation (SD), and were compared between groups using one-way ANOVA. Categorical variables were presented as frequencies or percentages, and unadjusted comparisons between the LPA and HPA groups were performed using chi-squared (χ2) tests.

Then, we performed the generalized linear models (GLM) to examine the relationships between the WGRS, PA levels, and their interaction effects on BMI and body weight change. Stratified generalized linear models were applied to analyze longitudinal associations between the WGRS and changes in BMI/body weight from the first to fourth years of the natural experiment, with stratification based on PA levels. Effect sizes were expressed as β coefficients with corresponding 95% confidence interval (95% *CI*). All models were adjusted for age, sex, ethnicity, and dietary factors. Dietary factors were assessed using the self-reported FFQ, and exploratory factor analysis was used to generate dietary pattern factor score. Factor scores showing between-group differences were converted into trichotomous variables and included in the models as covariates. Statistical significance was set at *P* < 0.05. All statistical analyses were performed using SPSS 20.0 and STATA SE 12.0.

## Results

### Baseline characteristics of participants

A total of 591 participants were enrolled in this study, including 80 male students (13.5%) and 511 female students (86.5%). Based on their physical activity level, the participants were divided into the LPA group (*n* = 199) and the HPA group (*n* = 392). As shown in Table 1, significant differences were observed between the two groups in age, sex, height, weight, BMI, and several metabolic indices, including triglycerides, HDL-C, apolipoprotein A1, fasting blood glucose (FBG), creatinine, and uric acid.

**Table 1:** Demographic characteristics of baseline study participants

Variables	LPA	HPA	*P* value
**Age** (years)	12.49 ± 1.07	11.54 ± 1.24	< 0.001^*^
**Gender**			< 0.001^*^
Boys, n (%)	54 (27.1)	26 (6.6)	
Girls, n (%)	145 (72.9)	366 (93.4)	
**Ethnic**			0.09
Han, n (%)	176 (88.4)	326 (83.2)	
Others, n (%)	23 (11.6)	66 (16.8)	
**Anthropometry**			
Height (cm)	160.12 ± 9.64	151.25 ± 9.14	< 0.001^*^
Weight (kg)	52.87 ± 15.01	39.89 ± 7.03	< 0.001^*^
BMI (kg/m^2^)	20.33 ± 4.14	17.33 ± 1.86	< 0.001^*^
**Lipids**			
Triglyceride (mmol/L)	0.73 ± 0.39	0.50 ± 0.20	< 0.001^*^
Cholesterol (mmol/L)	3.98 ± 0.71	4.28 ± 0.75	0.002^*^
HDL-C (mmol/L)	1.42 ± 0.37	1.71 ± 0.34	< 0.001^*^
LDL-C (mmol/L)	2.09 ± 0.55	2.14 ± 0.62	0.54
Apo A1 (g/L)	1.44 ± 0.24	1.60 ± 0.22	< 0.001^*^
Apo B (g/L)	0.63 ± 0.15	0.64 ± 0.15	0.89
**Cardiometabolic markers**			
FBG (mmol/L)	4.73 ± 0.39	4.59 ± 0.34	0.002^*^
ALT (U/L)	18.28 ± 21.47	15.13 ± 12.78	0.14
AST (U/L)	27.16 ± 74.11	23.61 ± 23.65	0.55
Urea (mmol/L)	3.53 ± 0.85	3.44 ± 0.88	0.43
Creatinine (umol/L)	52.74 ± 9.84	48.22 ± 6.63	< 0.001^*^
Uric acid (umol/L)	362.07 ± 101.72	319.66 ± 73.79	< 0.001^*^

BMI, body mass index; FBG, fasting blood glucose; ALT, alanine aminotransferase; AST, aspartic aminotransferase; HDL-C, high-density lipoprotein cholesterol; LDL-C, low-density lipoprotein cholesterol

*P < 0.05

### Analysis of genetic susceptibility to obesity

**Fig. 2:**
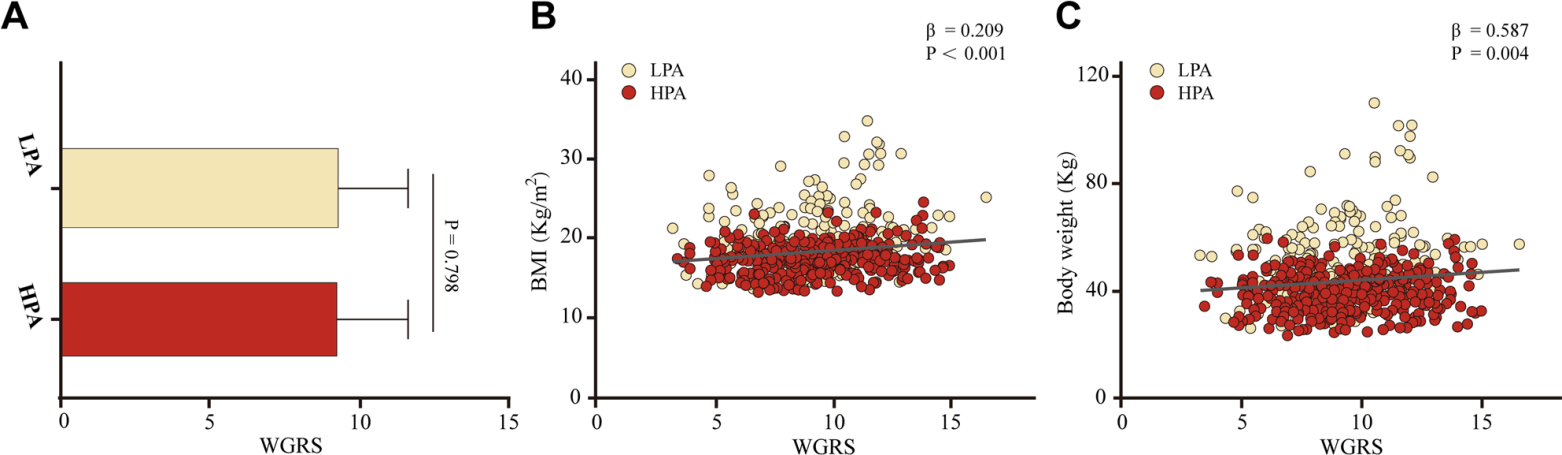
Assessment of genetic susceptibility to obesity.** A**. Weighted genetic risk score (WGRS) comparison between the low-PA (LPA) and high-PA (HPA) groups. Whiskers indicate the lowest and highest values within the mean ± standard deviation (SD); the long horizontal line within each whisker denotes the mean. **B**, **C**. Correlations between WGRS and body mass index (BMI) and between WGRS and body weight. Circles represent individual data points for each group

Based on the genotyping results for 13 SNP loci, the WGRS for obesity was calculated for each participant. No statistically significant difference in the WGRS was observed between group (*P* = 0.798, Fig. 2A). Linear regression analysis showed that the WGRS was positively associated with the BMI; for each 1-point increase in WGRS, the BMI increased by 0.209 kg/ m^2^ (95% *CI*: 0.10–0.32 kg/ m^2^, *P* < 0.001, Fig. 2B). As shown in Fig. 2C, the WGRS was also correlated with body weight (β = 0.587, *P* = 0.004). These results suggest that the WGRS, estimated from 13 obesity-related SNP loci, is an effective proxy for assessing genetic susceptibility to obesity.

### Assessment of PA levels and dietary patterns during the natural experiment period

To compare PA levels between groups during the natural experiment, the HBSC questionnaire was administered 1 year after the intervention. A total of 565 valid questionnaires were collected, including 187 from the LPA group and 378 from the HPA group. As shown in Figure S2, in the HPA group, 66.14% of participants reported achieving an average of ≥ 60 min of moderate-to-vigorous PA (MVPA) per day for 6–7 days per week, compared with only 10.70% in the LPA group (*P* < 0.001). Similarly, 59.52% of the HPA group reported engaging in vigorous PA daily, versus 28.88% in the LPA group (*P* < 0.001). Overall, self-reported data indicated significantly higher PA levels in the HPA group than in the LPA group during the natural experiment period.

To assess dietary pattern, it is important to note that all participants were boarding students sharing broadly similar living and dietary environments. Despite this homogeneity, dietary behaviors were evaluated using the FFQ. Among 530 valid responses, factor analysis identified five distinct dietary patterns (KMO = 0.89; Bartlett’s sphericity test *P* < 0.001), collectively explaining 60.91% of the total variance in dietary intake (see Additional file 1, Figure S3A). As shown in Figure S3B, factor score comparisons revealed significant intergroup differences in grain/meat-type and snack-type dietary patterns (*P* < 0.05).

### Trajectory of changes in obesity and related metabolic indicators

**Fig. 3:**
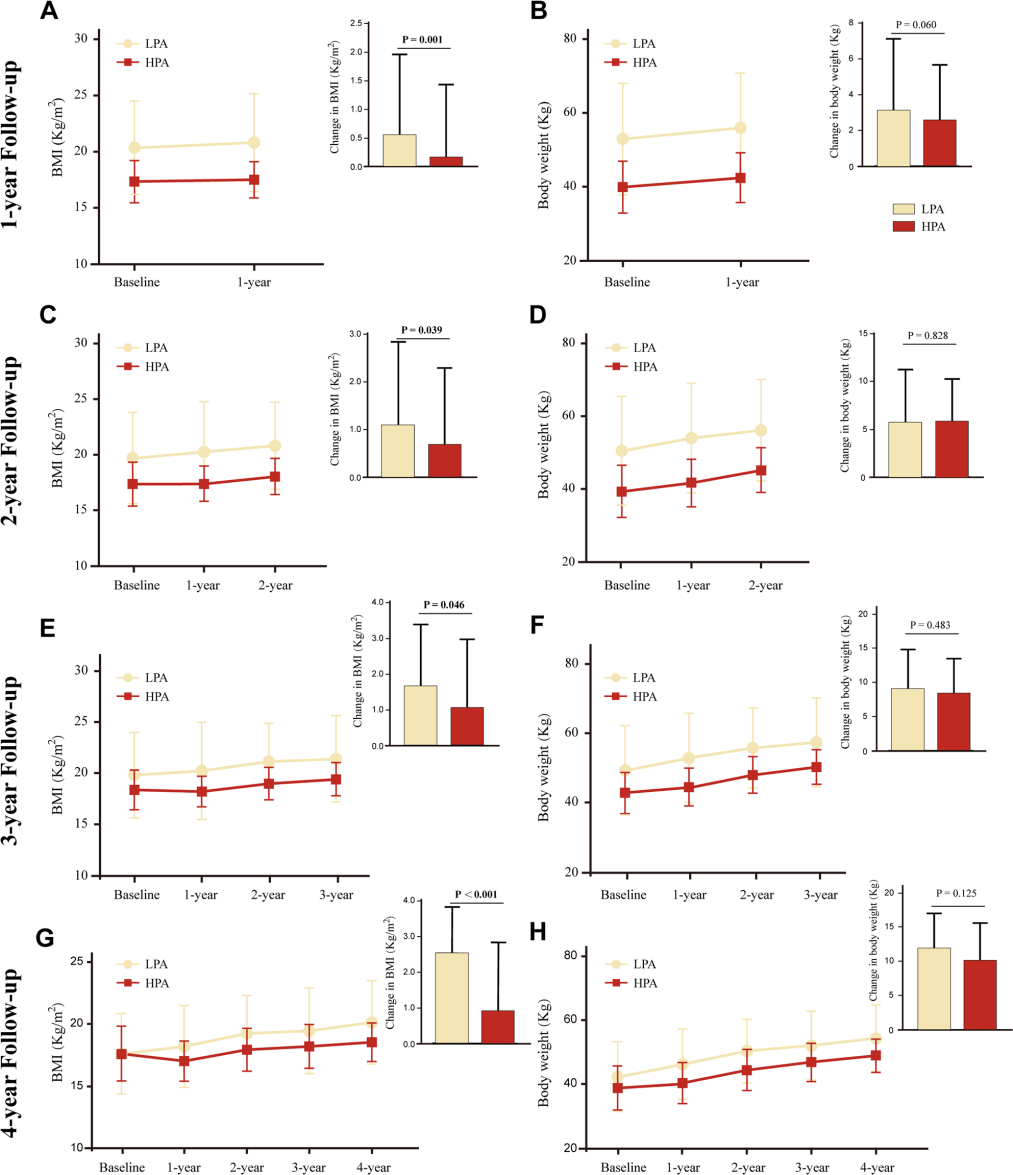
Assessment of BMI and body weight trajectories during the natural experiment period. **A**, **B**. Comparative analysis of BMI and body weight change trajectories and change amounts between the LPA and HPA groups during the 1-year follow-up period. **C**, **D**. Comparative analysis of BMI and body weight change trajectories and change amounts between the LPA and HPA groups during the 2-year follow-up period. **E**, **F**. Comparative analysis of BMI and body weight change trajectories and change amounts between the LPA and HPA groups during the 3-year follow-up period. **G**, **H**. Comparative analysis of BMI and body weight change trajectories and change amounts between the LPA and HPA groups during the 4-year follow-up period. Whiskers indicate the lowest and highest values within the mean ± SD; the long horizontal line within each whisker denotes the mean

As shown in Fig. 3, overall, both the LPA and HPA groups showed increases in BMI and body weight over the 4 years of the natural experiment compared with baseline. From the first to fourth year, the BMI increases in the HPA group were 0.39 kg/m^2^, 0.40 kg/m^2^, 0.61 kg/m^2^, and 1.61 kg/m^2^ lower, respectively, than those in the LPA group. For body weight, the HPA group also showed lower average increases than the LPA group, although these differences were not statistically significant. Regarding fasting blood glucose (see Additional file 1, Figure S4A), the HPA and LPA groups decreased by 0.16 mmol/L and 0.31 mmol/L, respectively, with the LPA group showing a significantly greater reduction (*P* = 0.004).

### Impact of gene–PA interaction on obesity and related metabolic indicators

**Fig. 4:**
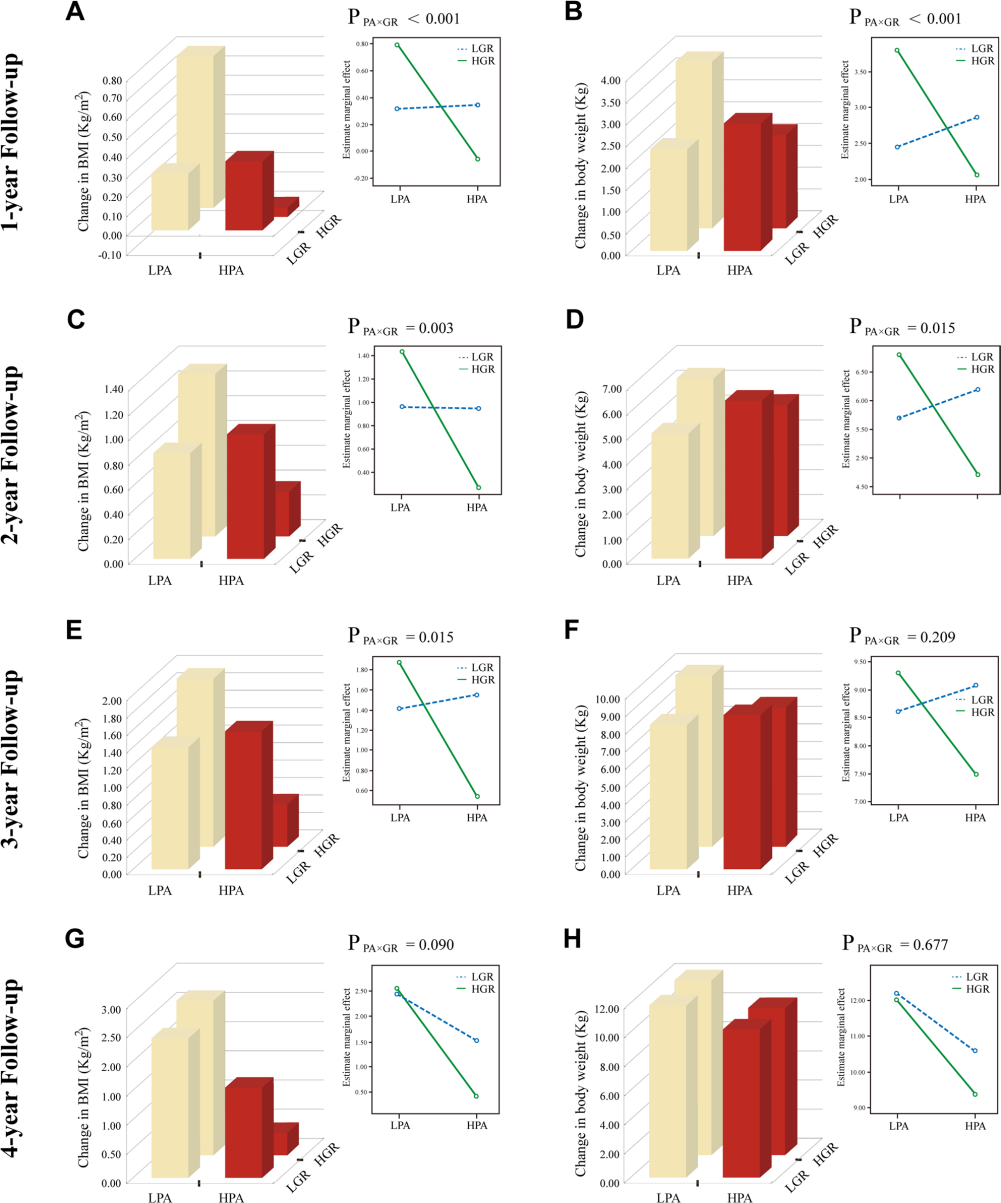
Gene–PA interaction effects on BMI and body weight. (**A**) Intergroup differences in BMI changes and gene–PA interaction effects on BMI during the 1-year follow-up period. (**B**) Intergroup differences in body weight changes and gene–PA interaction effects on body weight during the 1-year follow-up period. (**C**) Intergroup differences in BMI changes and gene–PA interaction effects on BMI during the 2-year follow-up period. (**D**) Intergroup differences in body weight changes and gene–PA interaction effects on body weight during the 2-year follow-up period. (**E**) Intergroup differences in BMI changes and gene–PA interaction effects on BMI during the 3-year follow-up period. (**F**) Intergroup differences in body weight changes and gene–PA interaction effects on body weight during the 3-year follow-up period. (**G**) Intergroup differences in BMI changes and gene–PA interaction effects on BMI during the 4-year follow-up period. (**H**) Intergroup differences in body weight changes and gene–PA interaction effects on body weight during the 4-year follow-up period. All analyses were adjusted for age, sex, ethnicity, and dietary patterns

Longitudinal analyses over 4 years of natural experimentation revealed dynamic gene–PA interactions affecting obesity and related outcomes. At the 1-year follow-up, participants with higher genetic susceptibility to obesity in the HPA group showed a greater decrease in BMI (0.36 ± 1.15 vs. −0.05 ± 1.37 kg/m^2^, *P* = 0.002) and body weight (2.89 ± 3.07 vs. 2.12 ± 3.18 kg, *P* = 0.02) than those with lower genetic susceptibility. After adjusting for age, sex, ethnicity, and dietary factors, there was a significant interaction between PA and the WGRS on changes in BMI and body weight (both *P* < 0.001, Fig. 4A-B). Additionally, the interaction between genetic susceptibility and PA for the fasting blood glucose and triglycerides was not statistically significant (*P* = 0.59 and 0.26, see Additional file 1, Figure S4C-D). This protective effect persisted at the 2- and 3-year follow-ups. In the HPA group, participants with higher genetic susceptibility continued to show significantly greater reductions in BMI compared to those with lower genetic susceptibility (*P* = 0.004 and 0.01, respectively), with significant gene–PA interactions observed for BMI change at both time points (*P* = 0.003 and 0.02, respectively). At the 4-year follow-up, in the HPA group, participants with a higher WGRS had a greater decrease in BMI (1.54 ± 1.49 vs. 0.37 ± 2.10 kg/m^2^, *P* = 0.03). Generalized linear model analysis indicated that interactions between PA and the WGRS for changes in BMI and body weight were not significant (*P* = 0.09 and 0.68). As hypothesized, the protective effect of PA on BMI gain was most pronounced among participants with higher genetic risk scores.

### Stratified regression analysis of the impact of gene–PA interaction on overweight and obesity

**Fig. 5:**
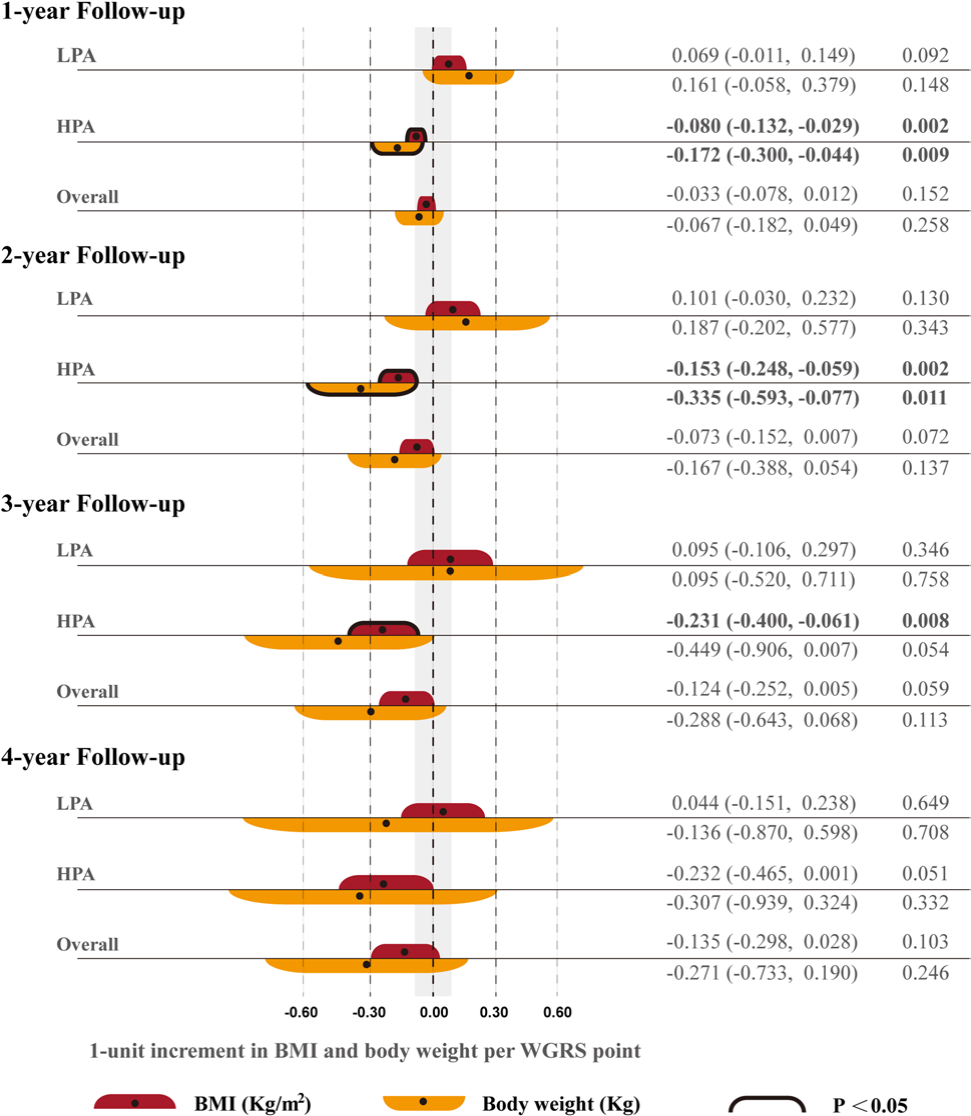
Stratified regression analysis of gene–PA interaction effects on BMI and body weight. Stratified by PA levels, association magnitudes are expressed as the change in BMI (kg/m^2^) or body weight (kg) per 1-point increment in the WGRS. All analyses were adjusted for age, sex, ethnicity, and dietary patterns. Colored dots represent β regression coefficients, colored shading indicates 95% confidence intervals, and boundaries on the shading denote *P* < 0.05

We established stratified linear regression models to assess quantitative gene–PA interaction effects on obesity and related metabolic markers. As shown in Fig. 5, after adjusting for age, sex, ethnicity, and dietary factors, each 1-unit increase in the WGRS in the HPA group was associated with a decrease of approximately 0.08 kg/m^2^ in BMI change and 0.17 kg in body weight change during the 1-year follow-up (*P* = 0.002 and 0.01, respectively), a decrease of approximately 0.153 kg/m^2^ in BMI change and 0.335 kg in body weight change during the 2-year follow-up (*P* = 0.002 and 0.01), a decrease of approximately 0.23 kg/m^2^ in BMI change during the 3-year follow-up (*P* = 0.01), and a decrease of approximately 0.23 kg/m^2^ in BMI change during the 4-year follow-up, which was at the margin of statistical significance (*P* = 0.05). By contrast, the regression coefficients between WGRS and changes in BMI and body weight in the LPA group were not statistically significant. These findings suggest that individuals with high genetic risk for obesity can benefit more from long-term high levels of PA.

## Discussion

In this study, we found that the WGRS—a weighted score of genetic susceptibility to obesity based on genotyping 13 obesity-related SNP loci—was associated with baseline BMI and body weight. Over the 1- to 4-year natural experiment period, the increase in BMI was smaller in the HPA group than in the LPA group. Multivariable models adjusting for age, sex, ethnicity, and dietary patterns identified significant PA–WGRS interaction effects on BMI changes. Participants with higher genetic risk scores for obesity experienced less BMI and weight gain during long-term high-intensity PA.

### Evaluation of long-term PA natural experiment outcomes

In this study, the participants were stratified into an HPA group consisting of dance students—a discipline classified as vigorous-intensity PA (4.5–7.3 METs) for youth—linked to improved cardiometabolic health, weight management, stress resilience, and reduced somatic symptoms [17]. Dance students generally engage in PA levels exceeding recommended guidelines [18]. A meta-analysis has shown that structured dance interventions improve body composition, blood biomarkers, and musculoskeletal function, with efficacy comparable to or exceeding that of other planned PA interventions [19]. By contrast, the LPA group included music, fine arts, and film students, whose PA levels were markedly lower than those of dance students. Previous studies have confirmed that music performance students engage in less PA than students in conventional majors [14]. Quantitatively, our data showed that 66.14% of the HPA group met or exceeded 60 min of MVPA per day for 6–7 days per week, compared with only 10.70% in the LPA group.

The natural divergence in activity levels across majors, combined with stable long-term curricular exposure, enabled a quasi-experimental design resembling a randomized controlled trial. While randomized controlled trials remain the gold standard for causal inference, they often face challenges related to cost, feasibility for long-term interventions, and ethical constraints in policy contexts [20]. This natural experiment design offers a cost-efficient alternative for investigating gene–PA interactions, overcoming these limitations while preserving ecological validity. Longitudinal follow-up data indicated smaller BMI increases in the HPA group, consistent with prior findings. For example, a 3-year follow-up at Guangxi Arts University reported improved physiological metrics and reduced body morphology indices among art students participating in dance classes and sports events [21]. Similarly, Snyder et al. [22] observed higher health-related quality of life in school athletic club participants, and Huang et al. [23]. found that a 10-week dance program increased MVPA and improved BMI in children.

Notably, both groups in the present study showed upward trajectories in BMI and body weight, in contrast to adult studies where PA interventions typically reduce BMI. This difference reflects the developmental physiology of BMI in youth, where interventions aim to slow the normal growth-related increases in adiposity rather than achieve an absolute reduction. Obesity genetic susceptibility exerts age-dependent effects on weight dynamics [9]. In our study, participants with higher genetic susceptibility to obesity in the LPA group had greater BMI increases only during the first follow-up year, with no significant associations observed in subsequent years. Linear regression models likewise found no association between WGRS and BMI or weight changes in the LPA group. Rukh et al. [7]. reported that a GRS based on 31 BMI-related SNPs predicted annual weight gain in young-to-middle-aged adults but inversely predicted weight change in older adults. Similarly, a Swedish aging cohort found that a GRS predicted BMI changes in early adulthood but not after middle age [6], supporting the concept of developmental windows for genetic susceptibility expression.

### Genetic susceptibility modulates obesity outcomes in long-term natural experiments

The interaction between PA and genetic susceptibility to obesity remains debated, with most evidence centered on the *FTO* gene, the first genome-wide significant obesity locus identified through GWAS [24]. A previous cohort study of 15,925 Swedish and 2,511 Finnish adults found no significant interaction between the *FTO* rs9939609 locus and PA levels on obesity-related outcomes [25]. And, a cohort study of Singapore, Chinese, and Malaysian populations showed that regular PA attenuated the BMI-increasing effect of the *FTO* rs9939609 risk allele; however, the interaction between PA and genotype on BMI did not reach statistical significance [26]. In addition, a meta-analysis further reported a significant interaction between *FTO* rs9939609 and PA on BMI in adults, with PA attenuating 27% of the obesity risk conferred by this genetic variant; however, such gene–PA interactions on BMI were not observed in youth populations [9]. Nevertheless, Rampersaud et al. [27]. identified significant interactions between *FTO* variants (rs1861868 and rs1477196) and PA levels on obesity risk. Vimaleswaran et al. [28]. subsequently reported, in a longitudinal cohort, that PA attenuates the effect of *FTO* rs1121980 on BMI and waist circumference. Ahmad et al. [29]. highlighted lifestyle modulation of *FTO* rs8050136 effects in 21,675 White women in the United States. Reddon et al. [30]., in a multiethnic prospective cohort spanning 21 countries (*n* = 126,443; mean follow-up 3.3 years), observed PA-mediated attenuation of *FTO* rs1421085 related obesity risk, with subsequent replication in African- and European-American men [31]. And, a meta-analysis of lifestyle interventions also confirmed greater weight loss among carriers of the *FTO* rs9939609 risk allele [32]. Collectively, these findings position PA as a precision strategy for mitigating polygenic risk, with effect sizes modulated by both genetic dosage and intervention intensity.

Leveraging nationally representative data from the 2010–2012 China Nutrition and Health Surveillance, a previous study showed that higher PA levels significantly attenuated the obesogenic effects of multiple obesity-associated SNPs—including *MC4R* rs12970134 and *FTO* rs9939609—on BMI, waist circumference, and central adiposity risk in Chinese adults [33]. Earlier work by Mi et al. [34] suggested interaction effects between *FTO rs9939609* and lifestyle factors (PA, diet, sedentary time) on obesity in Chinese youth, although the findings were limited by the cross-sectional design.

Chinese populations exhibit distinct genetic architecture at *FTO* loci compared with European-ancestry cohorts, characterized by significantly lower minor allele frequencies of obesity-associated SNPs. This difference reduces the predictive utility of *FTO* variants for obesity risk stratification in Chinese individuals, making polygenic risk scores essential for accurate genetic susceptibility assessment. In the present study, we constructed a WGRS integrating 13 obesity-associated SNPs to overcome the power limitations of single-variant analyses in populations with low minor allele frequencies. After adjusting for age, sex, ethnicity, and dietary confounders, multivariable models revealed significant PA–WGRS interactions on longitudinal BMI trajectories. Parallel findings have emerged from previous longitudinal cohorts. A large prospective cohort study of European-ancestry adults (mean follow-up 3.6 years) found a significant interaction between a WGRS based on 12 obesity-susceptibility SNPs and PA levels on BMI trajectories, with sustained PA engagement attenuating 40% of the genetically driven obesity risk [35]. Franks et al. [36]. further linked specific SNPs (e.g., *PPARG* Pro12Ala, *LYPLAL1* rs2605100, *GNPDA2* rs10938397, *MTCH2* rs10838738, *NEGR1* rs2815752 and *FTO* rs9939609) to differential weight loss responses in diabetes prevention programs. Sedentary behavior has also been validated as a modifier of genetic susceptibility to obesity. In a previous study, analysis of 10-year longitudinal data from the NHS and HPFS cohorts revealed significant interactions between a 32-SNP-based GRS for BMI and both sedentary time and PA levels on BMI trajectories. Each 1-hour/day increase in screen-based sedentary time amplified the GRS–BMI association, whereas leisure-time PA attenuated this relationship [37]. In addition, prior evidence from the NHS and HPFS cohorts conducted a 20-year longitudinal analysis of gene–PA interactions on obesity and related metabolic outcomes [10]. Notably, in that study, which utilized a GRS incorporating 77 BMI-associated and 12 body fat-associated SNPs, a significant interaction between genetic susceptibility and PA levels on long-term BMI trajectories was reported. Specifically, for each 10-risk allele increment, high-PA individuals showed a BMI reduction of − 0.02 kg/m^2^ over 4 years, whereas low-PA individuals experienced a BMI increase of + 0.24 kg/m^2^. In our study, after adjusting for potential confounders, we observed that at 1 to 4 years of the natural experiment period, each 1-unit increase in the WGRS in the HPA group was associated with a decrease of approximately 0.08 kg/m^2^, 0.153 kg/m^2^, 0.23 kg/m^2^, and 0.23 kg/m^2^ in BMI changes, respectively. Collectively, existing evidence and our results suggest that the individual effect of each obesity-related genetic locus on BMI change in response to PA is modest, and thus may have limited clinical relevance at the individual level. However, at the population level, if genetic susceptibility data can be utilized to identify and distinguish individuals based on their responsiveness to PA intervention, this information could hold significant public health value for developing precise intervention strategies.

### Limitations

This study has several possible limitations. First, PA levels during the natural experiment were assessed using self-reported questionnaires rather than objective measures such as triaxial accelerometers (e.g., ActiGraph GT3X+), introducing the potential for self-report bias. Second, obesity evaluation relied primarily on BMI and body weight, with limited inclusion of glucose and lipid profiles, and it did not incorporate comprehensive assessments of body fat distribution or other multidimensional adiposity indicators. Third, the study did not investigate potential gene–gene interactions in modulating the effects of long-term PA interventions. Fourth, participants were exclusively students in art-related disciplines; thus, the generalizability of these findings should be interpreted with caution. Future validation through rigorously designed population-based studies with broader demographic representation is warranted. Fifth, given the unique characteristics of arts education, students enrolled in high-PA disciplines had significantly lower BMI levels at baseline than those in low-PA disciplines, with notable sex-based disparities in discipline selection. Prior studies have shown that individuals with higher baseline body weight or BMI tend to exhibit greater responsiveness to weight loss interventions than do those with lower BMI [38, 39]. Sixth, dietary intake was assessed using a non-quantitative FFQ, which does not capture absolute caloric intake or precise macronutrient composition.

## Conclusions

In summary, the present study demonstrated a significant correlation between the WGRS for obesity predisposition and both baseline BMI and body weight. During the natural experiment period, the HPA group showed a smaller BMI increase than the LPA group, although no statistically significant between-group differences were observed for body weight changes. In longitudinal analyses adjusted for age, sex, ethnicity, and dietary factors, we identified a significant interaction effect between PA levels and WGRS in modifying BMI trajectories. Collectively, these findings underscore that sustained PA can modulate genetic susceptibility to obesity. Identifying genetically high-risk individuals who are responsive to PA-based interventions holds substantial potential for disease prevention and supports the development of personalized obesity management strategies.

## Supplementary Information

Below is the link to the electronic supplementary material.

Supplementary Material 1

Supplementary Material 2

## Data Availability

The datasets used and/or analysed during the current study are available from the corresponding author on reasonable request.

## Abbreviations

PA: Physical activity
WGRS: Weighted genetic risk score
NHS: Nurses’ Health Study
METs: Metabolic equivalents
FFQ: Food frequency questionnaire
SD: Standard deviation
FBG: Fasting blood glucose
BMI: Body mass index
SNP: Single-nucleotide polymorphisms
HPFS: Health Professional Follow-Up Study
HBSC: Health Behaviors in School-aged Children
GWAS: Genome-wide association study
GLM: Generalized linear models
MVPA: Moderate-to-vigorous PA
