# Supplementary material for: Obesity, physical activity, and gene–environment interactions: a natural experiment framework
Source: J Health Popul Nutr. 2026 Apr 9;45:149. doi: 10.1186/s41043-026-01312-y (PMC13262356; doi:10.1186/s41043-026-01312-y)

* Supporting Information

**Obesity, Physical Activity, and Gene–Environment Interactions: A Natural Experiment Framework**

Running Title: Gene and physical activity interaction on obesity

**Authors**

Lei Liu^1,2,3,4^, Xuxiu Zhuang^1,3^, Haonan Zhou^1,3^, Yanan Ma^1,3^, Gengrun Sun^2,4^, Yang Liu^1,3*^, Deliang Wen^1,3*^

**Affiliations**

1. Health Sciences Institute, China Medical University, Shenyang, Liaoning Province 110122, PR China
2. School of Life Sciences and Health, University of Health and Rehabilitation Sciences, Qingdao, Shandong Province 266113, PR China
3. Key Laboratory of Obesity and Glucose/Lipid Associated Metabolic Diseases, China Medical University, Shenyang, Liaoning Province 110122, PR China
4. Endocrinology Department, Qingdao Hospital, University of Health and Rehabilitation Sciences (Qingdao Municipal Hospital), Qingdao, Shandong Province 266113, PR China

***Corresponding authors**

Correspondence: Yang Liu, Health Sciences Institute, China Medical University, Shenyang, Liaoning Province, China (E-mail: yliu0568@cmu.edu.cn); Deliang Wen, Health Sciences Institute, China Medical University, Shenyang, Liaoning Province, China (E-mail: dlwen@cmu.edu.cn)

Table S1 Classification of overweight and obesity in Chinese children and adolescents

| Age | Boys | | |  | Girls | | |
| --- | --- | --- | --- | --- | --- | --- | --- |
|  | Normal | Overweight | Obesity |  | Normal | Overweight | Obesity |
| 6 | <16.6 | 16.6-18.1 | ≥18.1 |  | <16.3 | 16.3-17.9 | ≥17.9 |
| 7 | <17.4 | 17.4-19.2 | ≥19.2 |  | <17.2 | 17.2-18.9 | ≥18.9 |
| 8 | <18.1 | 18.1-20.3 | ≥20.3 |  | <18.1 | 18.1-19.9 | ≥19.9 |
| 9 | <18.9 | 18.9-21.4 | ≥21.4 |  | <19.0 | 19.0-21.0 | ≥21.0 |
| 10 | <19.6 | 19.6-22.5 | ≥22.5 |  | <20.0 | 20.0-22.1 | ≥22.1 |
| 11 | <20.3 | 20.3-23.6 | ≥23.6 |  | <21.1 | 21.1-23.3 | ≥23.3 |
| 12 | <21.0 | 21.0-24.7 | ≥24.7 |  | <21.9 | 21.9-24.5 | ≥24.5 |
| 13 | <21.9 | 21.9-25.7 | ≥25.7 |  | <22.6 | 22.6-25.6 | ≥25.6 |
| 14 | <22.6 | 22.6-26.4 | ≥26.4 |  | <23.0 | 23.0-26.3 | ≥26.3 |
| 15 | <23.1 | 23.1-26.9 | ≥26.9 |  | <23.4 | 23.4-26.9 | ≥26.9 |
| 16 | <23.5 | 23.5-27.4 | ≥27.4 |  | <23.7 | 23.7-27.4 | ≥27.4 |
| 17 | <23.8 | 23.8-27.8 | ≥27.8 |  | <23.8 | 23.8-27.7 | ≥27.7 |
| 18 | <24.0 | 24.0-28.0 | ≥28.0 |  | <24.0 | 24.0-28.0 | ≥28.0 |

**References:** Group of China Obesity Task Force. [Body mass index reference norm for screening overweight and obesity in Chinese children and adolescents]. *Zhonghua Liu Xing Bing Xue Za Zhi*. 2004 Feb;25(2):97-102. PMID: 15132858.

Table S2 The 24 food categories included in the non-quantitative food frequency questionnaire (FFQ)

| Category No. | Food Category | Examples of Included Food Items |
| --- | --- | --- |
| 1 | Grains (whole grains) | White rice, brown rice |
| 2 | Flour and/or rice products | Noodles, steamed buns, pancakes |
| 3 | Livestock meat | Pork, beef, lamb, mutton |
| 4 | Poultry | Chicken, duck, goose |
| 5 | Fish | Carp, grass carp, crucian carp, sea fish |
| 6 | Other seafood | Shrimp, shellfish, squid, cuttlefish, octopus |
| 7 | Fresh vegetables | Spinach, kale, potatoes, taro, carrots |
| 8 | Fresh fruit (excluding fruit juice) | Apples, bananas, oranges, grapes, watermelon |
| 9 | Eggs | Chicken eggs, duck eggs, quail eggs |
| 10 | Milk | Fresh milk |
| 11 | Dairy products | Cheese, yogurt |
| 12 | Soy products | Soybeans, tofu, dried tofu, soy milk |
| 13 | Candy and chocolate | Fruit candies, jelly, hard candies, chocolate |
| 14 | Nuts and seeds | Peanuts, cashews, walnuts, almonds, chestnuts, pistachios, hazelnuts, melon seeds, sesame seeds |
| 15 | Dried and preserved fruits | Raisins, dried plums |
| 16 | Puffed snacks | Rice crackers, other puffed cereal snacks |
| 17 | Potato chips or other chips | Potato chips, tortilla chips |
| 18 | Pastries or baked goods | Cakes, cookies, pies, tarts, sweet rolls, bread |
| 19 | Regular (sugar‑sweetened) beverages | Soda, Fanta, other carbonated soft drinks |
| 20 | Sugar‑free beverages | Diet Coke, Coke Zero |
| 21 | Fruit juice | Orange juice, coconut juice, apple juice |
| 22 | Functional beverages | Sports drinks, vitamin‑enhanced water, energy drinks |
| 23 | Tea | Black tea, green tea, bottled tea drinks |
| 24 | Other beverages | Lactic acid drinks |

Table S3 Summary of the BMI associated locus and genotyped SNPs

| Locus | Lead SNP | Genomic  Coordinate | Effect/  Other  Alleles | EAF | Beta of  Metabolite Level  per Allele | Call Rate |
| --- | --- | --- | --- | --- | --- | --- |
| *FTO* | rs17817449 | 16:53779455 | G/T | 11.6% | 0.079 | 99.3% |
| *MC4R* | rs6567160 | 18:60161902 | C/T | 22.3% | 0.051 | 99.7% |
| *GNPDA2* | rs10938397 | 4:45180510 | G/A | 28.8% | 0.37 | 98.8% |
| *BDNF* | rs6265 | 11:27658369 | C/T | 51.9% | 0.05 | 99.5% |
| *SEC16B* | rs574367 | 1:177904075 | T/G | 22.3% | 0.059 | 99.7% |
| *MAP2K5* | rs4776970 | 15:67788548 | A/T | 21.6% | 0.025 | 99.5% |
| *GIPR-QPCTL* | rs11671664 | 19:45669020 | G/A | 27.7% | 0.042 | 99.5% |
| *ADCY3-DNAJC27* | rs6545814 | 2: 24908447 | G/A | 41.5% | 0.033 | 98.5% |
| *CDKAL1* | rs9356744 | 6:20685255 | T/C | 15.4% | 0.034 | 99.5% |
| *PCSK1* | rs261967 | 5:96514546 | C/A | 43.1% | 0.038 | 98.8% |
| *GP2* | rs12597579 | 16:20246545 | C/T | 70.3% | 0.041 | 99.5% |
| *PAX6* | rs652722 | 11:31883988 | C/T | 63.9% | 0.037 | 99.5% |
| *SMC5-KLF9* | rs11142387 | 9:70383416 | C/A | 31.5% | 0.088 | 99.5% |

SNP, single nucleotide polymorphism; EAF, effect allele frequency.

Figure S1 Flow Diagram of Participant Enrollment


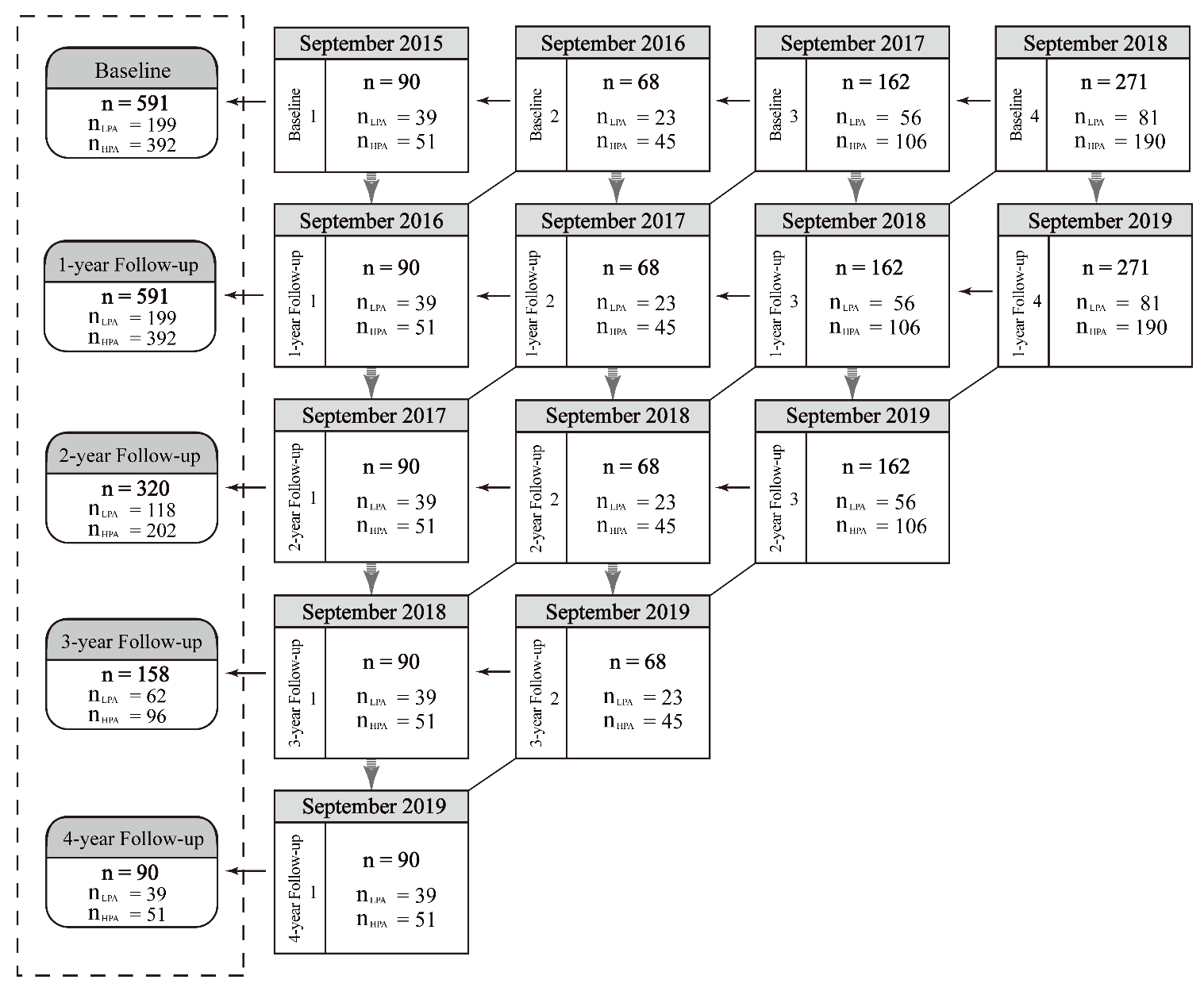


Figure S2 Comparative assessment of physical activity (PA) levels based on self-reported responses to the Health Behaviour in School-aged Children (HBSC) questionnaire.


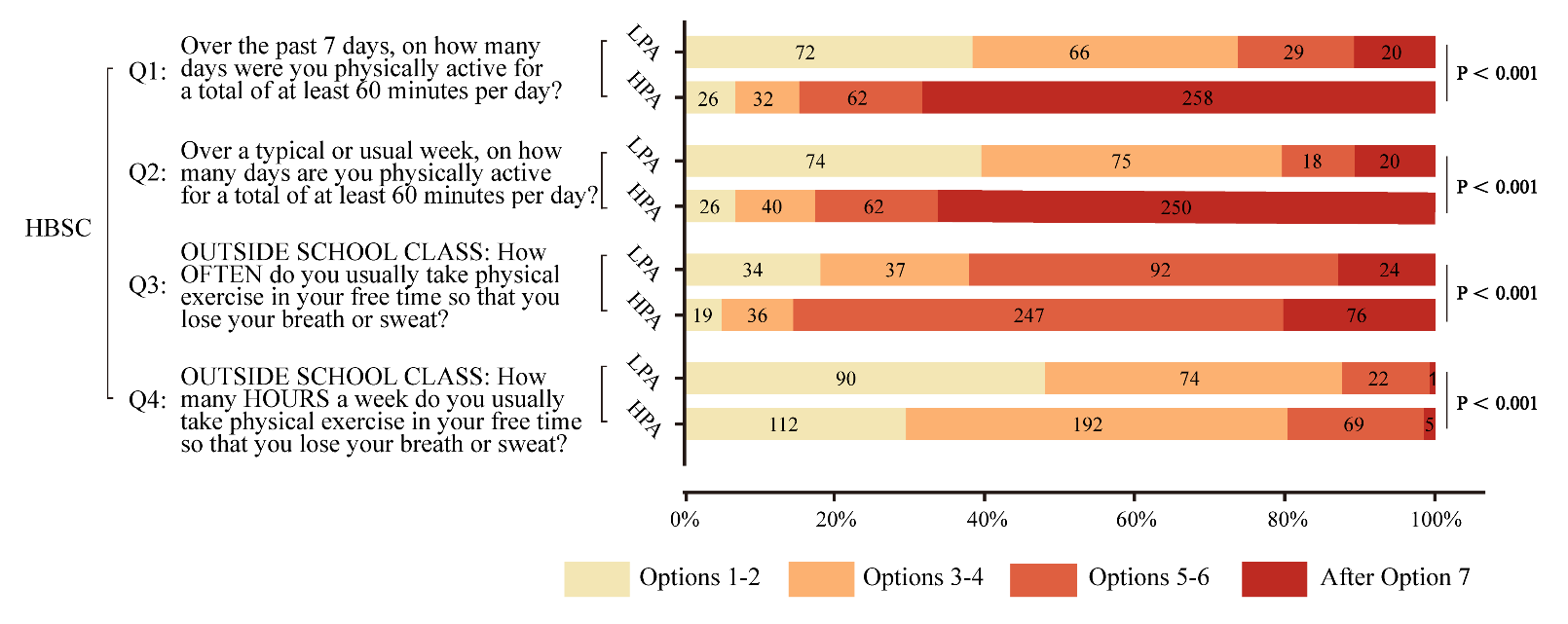


**Figure legends**:

(1) Questions 1 and 2: options 1–2 correspond to 0–1 days/week; options 3–4 correspond to 2–3 days/week. options 5–6 correspond to 4–5 days/week; option 7 corresponds to 6–7 days/week.

(2) Question 3: options 1–2 represent "Never" or "Less than once a month"; options 3–4 represent "Once a month" or "Once a week"; options 5–6 represent "2–3 times a week" or "4–6 times a week"; option 7 represents "Daily".

(3) Question 4: options 1–2 represent approximately 0–0.5 hours; options 3–4 represent approximately 1–3 hours; options 5–6 represent approximately 4–6 hours; option 7 represents ≥7 hours. All P-values were derived from chi-squared tests.

Figure S3 Dietary Pattern Assessment with Intergroup Comparison During a Natural Experiment


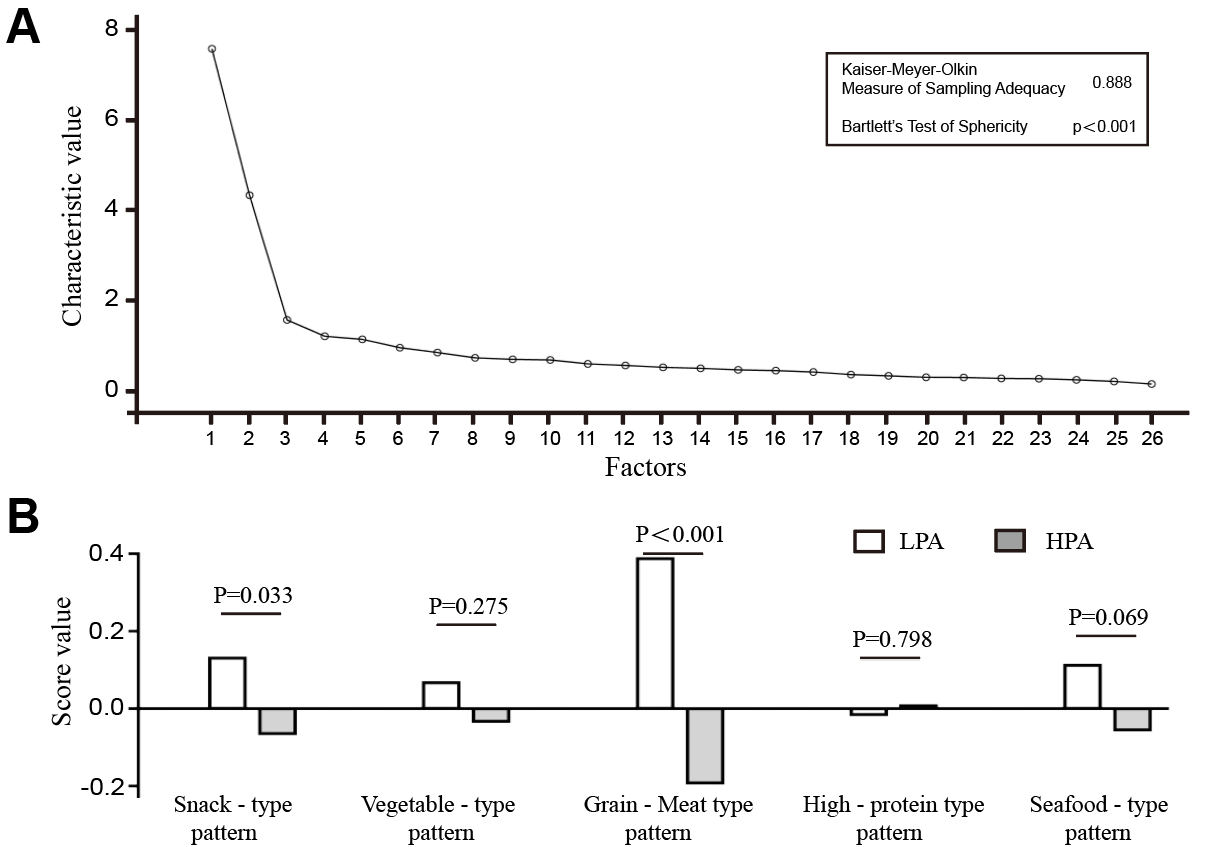


A: Scree plot; B: Intergroup comparison of dietary pattern scores

Figure S4 Gene**–**PA interaction effects on fasting blood glucose and cholesterol during one-year Follow-up


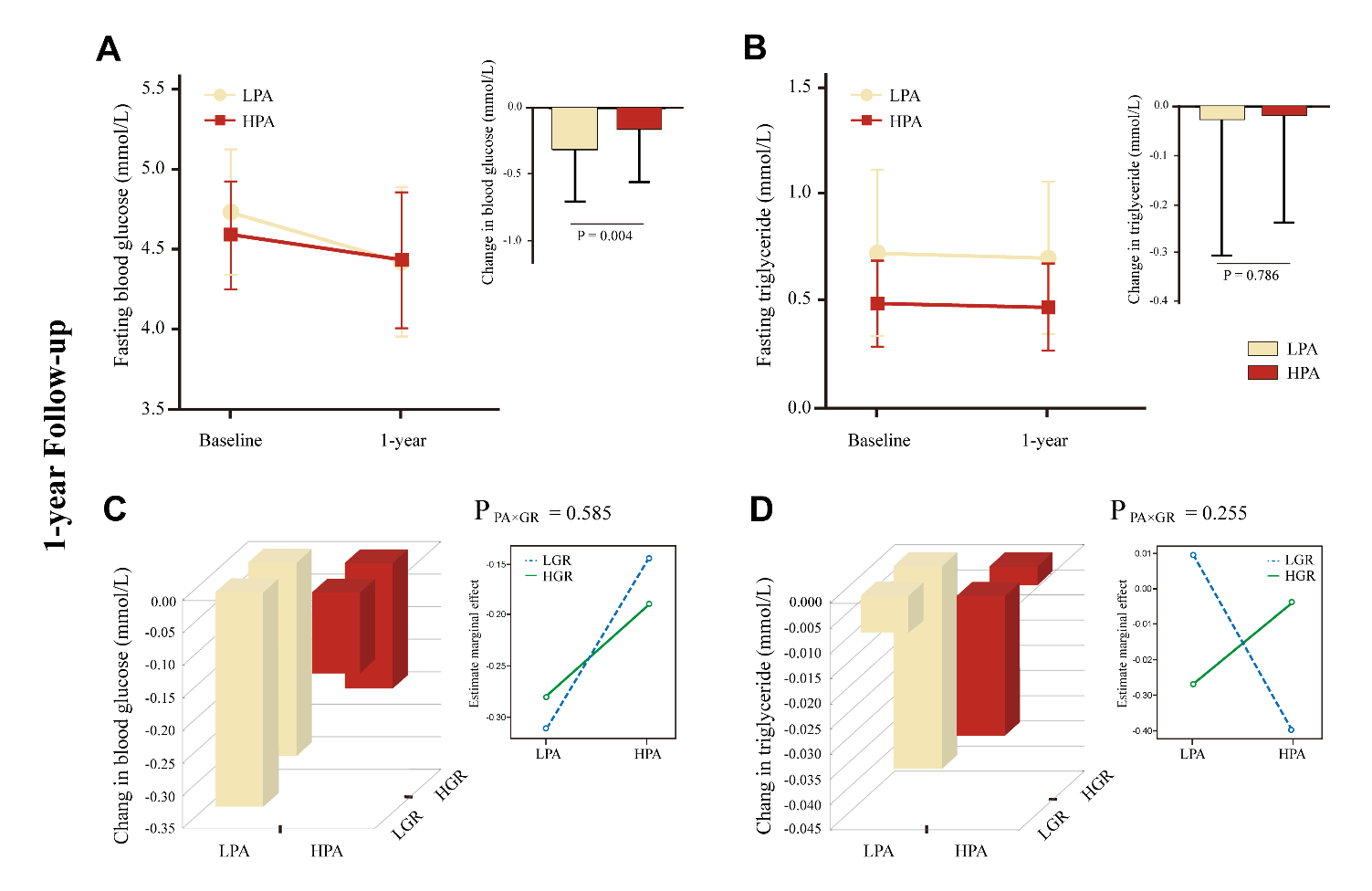

Supplement: Supplementary file 1 — Supplementary Material 1 [file 41043_2026_1312_MOESM1_ESM.docx]
